# Conformational and Molecular Structures of α,β-Unsaturated Acrylonitrile Derivatives: Photophysical Properties and Their Frontier Orbitals

**DOI:** 10.3390/molecules21040389

**Published:** 2016-03-28

**Authors:** María Judith Percino, Margarita Cerón, Oscar Rodríguez, Guillermo Soriano-Moro, María Eugenia Castro, Víctor M. Chapela, Maxime A. Siegler, Enrique Pérez-Gutiérrez

**Affiliations:** 1 Laboratorio de Polímeros, Centro de Química, Instituto de Ciencias, Benemérita Universidad Autónoma de Puebla (BUAP), Complejo de Ciencias, ICUAP, Edif. 103H, 22 Sur y San Claudio, C.P. 72570 Puebla, Puebla, Mexico; margarita.ceron@correo.buap.mx (M.C.); oscar.biome@gmail.com (O.R.); jesus.soriano@correo.buap.mx (G.S.-M.); mareug.castro@correo.buap.mx (M.E.C.); victor.chapela@correo.buap.mx (V.M.C.); 2 Department of Chemistry, Johns Hopkins University, New Chemistry Building, 3400 N. Charles St. Baltimore, MD 21218, USA; xray@jhu.edu; 3 Centro de Investigaciones en Óptica (CIO), Loma del Bosque 115, Lomas del Campestre, León, Guanajuato 37000, Mexico; eperez@cio.mx

**Keywords:** structure-property relationships, electrochemical properties, charge transfer, supramolecular network, D-π-A type dyes and DFT calculations

## Abstract

We report single crystal X-ray diffraction (hereafter, SCXRD) analyses of derivatives featuring the electron-donor *N*-ethylcarbazole or the (4-diphenylamino)phenyl moieties associated with a -CN group attached to a double bond. The compounds are (*2Z*)-3-(4-(diphenylamino)-phenyl)-2-(pyridin-3-yl)prop-2-enenitrile (**I**), (2*Z*)-3-(4-(diphenylamino)phenyl)-2-(pyridin-4-yl)-prop-2-enenitrile (**II**) and (*2Z*)-3-(9-ethyl-9*H*-carbazol-3-yl)-2-(pyridin-2-yl)enenitrile (**III**). SCXRD analyses reveal that **I** and **III** crystallize in the monoclinic space groups *P2/c* with *Z’* = 2 and *C2/c* with *Z’* = 1, respectively. Compound **II** crystallized in the orthorhombic space group *Pbcn* with *Z’* = 1. The molecular packing analysis was conducted to examine the pyridine core effect, depending on the *ortho*, *meta*- and *para*-positions of the nitrogen atom, with respect to the optical properties and number of independent molecules (*Z*’). It is found that the double bond bearing a diphenylamino moiety introduced properties to exhibit a strong π-π-interaction in the solid state. The compounds were examined to evaluate the effects of solvent polarity, the role of the molecular structure, and the molecular interactions on their self-assembly behaviors. Compound **I** crystallized with a cell with two conformers, *anti* and *syn*, due to interaction with solvent. DFT calculations indicated the *anti* and *syn* structures of I are energetically stable (less than 1 eV). Also electrochemical and photophysical properties of the compounds were investigated, as well as the determination of optimization calculations in gas and different solvent (chloroform, cyclohexane, methanol, ethanol, tetrahydrofuran, dichloromethane and dimethyl sulfoxide) in the Gaussian09 program. The effect of solvent by PCM method was also investigated. The frontier HOMO and LUMO energies and gap energies are reported.

## 1. Introduction

The study of π-conjugated organic compounds has led to the development of a rich variety of new concepts based on the interplay between their π-electronic structures and their molecular structures. These structures are associated with intense optical properties and thus, the identification and understanding of the structure-property relationships of these molecules is of key importance [1,2,3]. Bulk properties such as luminescence, excitation migration, and carrier mobility depend on the intermolecular dipole coupling, which is determined by the relative positions of adjacent molecules and the directions of their dipole moments [4,5,6,7,8,9,10,11,12,13,14]. Therefore, the design and synthesis of functional molecular solid-state structures or arrangements through tuning the intermolecular interactions remain challenging. The molecular arrangement of a compound in the solid state (*i.e*., the crystal packing) plays an important role in the performance of organic electronic devices [15,16,17,18].

Of the four main packing motifs in organic solid states [19,20,21,22,23,24,25], the typical herringbone packing, characterized by non-π-π overlapping of neighboring molecules, is unfavorable for charge transport. The large angle between the planes of adjacent molecules along the herringbone diagonal tends to reduce the strength of intermolecular interactions [26,27]. The non-classical herringbone packing with π-π overlap between neighboring molecules, also called slipped π-stacking, lamellar packing with one-dimensional (1-D) π-stacking, and lamellar packing with two-dimensional (2-D) π-stacking represent the other common forms of molecular packing. We have focused our investigations on the synthesis of different α,β-unsaturated acrylonitrile with different derivatives functionalized with electron donors such as diphenylamino-, dimethylamino-, and carbazole attached to the conjugated double bond and with electron acceptors such as the CN and the pyridyl core (in positions 4, 3, 2). Our goal was to observe the effect of substituent position on the optical properties [28,29,30,31]. We reported marked differences in the optical properties of the diphenylamino *versus* dimethylamine groups, specifically in their fluorescence behaviors, which we compared in the solid state, in single crystals, and in solution in different solvents [28,29]. For some organic materials, the fluorescence of the chromophore is quenched in the solid state, based on the phenomenon of aggregation-caused quenching (ACQ). In contrast, for other compounds, the emission depends on the formation of aggregates, termed aggregation-induced emission (AIE) or aggregation-induced emission enhancement (AIEE) [32,33,34,35,36,37]. The organic materials exhibit either no or weak fluorescence in solution and the aggregated particles (obtained by adding the solution into a poorly solubilizing solvent) exhibit relatively intense fluorescence upon UV irradiation such as for (dimethylamino)acrylonitrile derivatives, specifically the *Z*-2-(phenyl)-3-(4-dimethylaminophenyl)acrylonitrile [28] and α-cyano-stilbene [38].

Attempts are made to grow adequately sized single crystals in order to investigate that the types of cofacial packing led to different motifs in the herringbone packing. Several research groups have reported [25,26] that in the absence of electrostatic repulsion, organic molecules can be exactly superimposed on top of one another in a perfect co-facial situation, and usually any displacements can occur along the shortest/longest molecular axes between adjacent molecules. These theoretical investigations show that such displacements could strongly affect the intermolecular electronic couplings, in a way that depends on the bonding-antibonding pattern of the frontier molecular orbitals (HOMO, highest occupied molecular orbital, or LUMO, lowest unoccupied molecular orbital) [39]. These studies suggest that changes in the crystal packing were responsible for the crystallochromic property, *i.e.*, changes in the color of the crystal [40,41]. Crystal packing of a given structure is described by the unit cell and the crystallographic symmetry operations of the given space group. In other studies [42,43,44], the packing problem for high *Z’* structures (*i.e.*, structures with *Z’* > 1) is related to polymorphism for about 9% of crystal structures, although some chemically identical molecules are not related to one another by crystallographic symmetry and occupy distinctly independent positions. An examination of crystal packing is fundamental to investigate properties such as color and emission.

**Figure 1:**
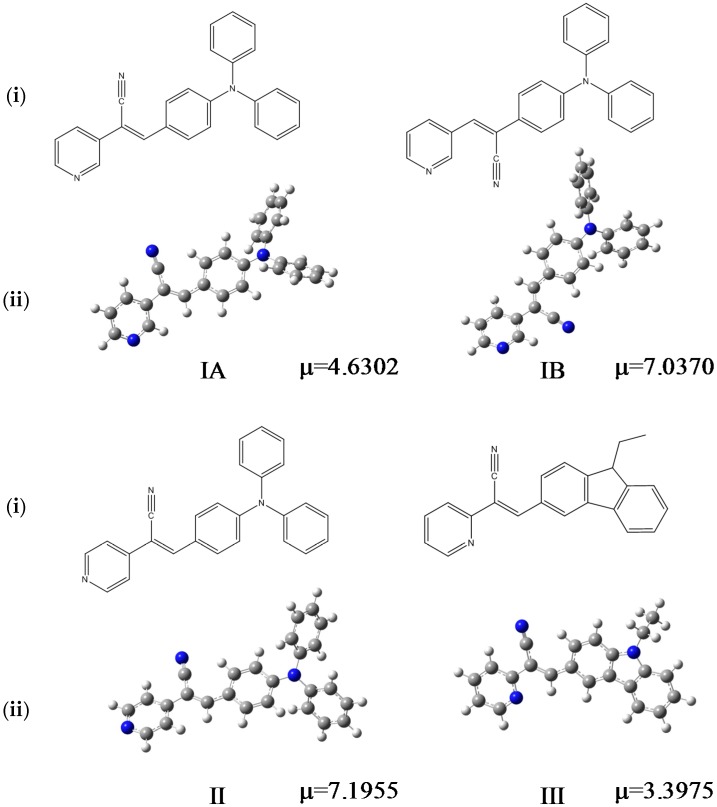
Chemical formulas of the compounds performed in ChemDraw software (**i**), optimized structures and µ (dipole moment, Debyes) calculated in gas phase at M06L/cc-pVDZ theory level (**ii**).

Understanding the nature of the interactions that determine the molecular packing in the solid state and how these interactions affect the optical and electrical properties of these materials is therefore essential for tuning their properties. It is well known that triphenylamine-based materials exhibit a variety of intra- and intermolecular interactions such as Van der Waals interactions, weak hydrogen bonding, π-π stacking, and nitrogen-CN interactions originating from the high polarizability of the CN electrons in the rings. Therefore, in the present study we rely on single crystal X-ray crystallography analyses to investigate different optical properties such as absorbance and fluorescence from derivatives with D-π-A type dyes (D = donor, A = acceptor) and the main packing motifs in organic solid states. The derivatives are (*2Z*)-3-(4-(diphenylamino)phenyl)-2-(pyridin-3-yl)prop-2-enenitrile, (2*Z*)-3-(4-(diphenylamino)phenyl)-2-(pyridin-4-yl)prop-2-enenitrile and (*2Z*)-3-(9-ethyl-9*H*-carbazol-3-yl)-2-(pyridin-2-yl)enenitrile (Figure 1). The compounds bear an α,β-unsaturated nitrile and a pyridine group in the *meta* (**I**), *para* (**II**) and *ortho* (**III**) positions, respectively. We report the influence of solvent polarity and the roles of the molecular structure and the molecular interactions on their self-assembly behaviors and their optical properties. We also performed theoretical calculations in different solvent (chloroform, cyclohexane, methanol, ethanol, tetrahydrofuran, dichloromethane and dimethyl sulfoxide) using the Gaussian09 program. DFT calculations were made to determine the frontier HOMO and LUMO energies and gap energies.

## 2. Results and Discussion

### 2.1. X-ray Crystallography and Photolysis Properties of ***I***–***III***

Crystallographic data for **I**–**III** are summarized in Table 1. For compounds **I**–**III** the data were collected at 110(2) K after the crystals had been flash-cooled from room temperature. The crystal structure of **I** (Figure 1) shows that the molecule of the *meta* position of the pyridyl ring (structure **I**) belongs to a monoclinic crystal with *Z′* > 2 independent molecules of **I** per asymmetric unit, which could be attributed to a co-crystal with the solvent, (eight per unit cell, *Z* = 8) and to the *P2/c* space group. Crystal **II** belongs to an orthorhombic system (*Pbcn*, *Z* = 8). Crystal **III** also belongs to a monoclinic system with space group *C2/c* with a *Z* = 8, indicating an ordered structure. Both **II** and **III** display *Z′* = 1. Notably, for **I**, **II**, and **III**, the unit cell contained eight molecules.

**Figure 2:**
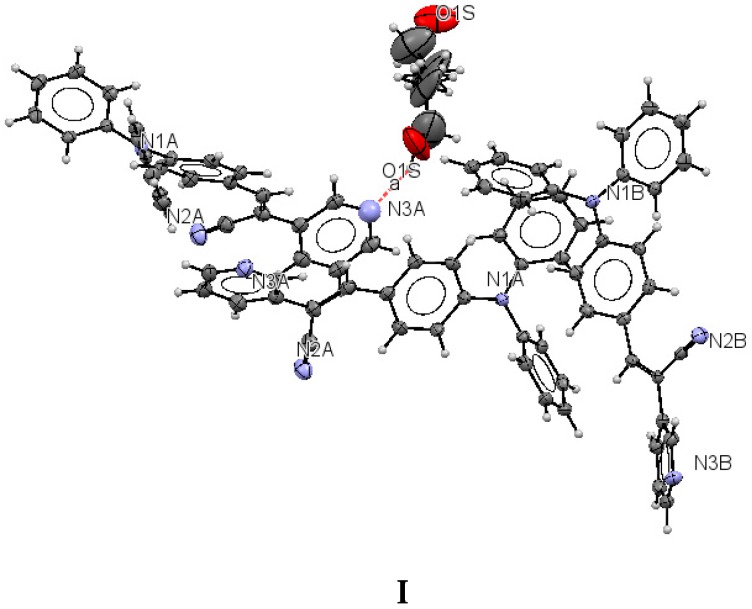

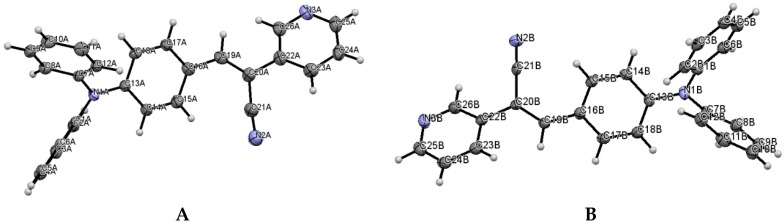
Displacement ellipsoid plots (50% probability level) of **I**. The two crystallographically independent molecules **A** and **B** are shown separately.

The structure of (**I**) is modeled as ordered even though the lattice ethanol molecules are likely to be disordered as they are located at sites of two-fold axis symmetry. The elongated ellipsoids found for the lattice solvent molecule (Figure 2) are consistent with this observation. The occupancy factor for the lattice solvent molecule was refined freely, and its value is 0.791(7). It is possible that the void contains a mixture of disordered solvent molecules. The twin relationship corresponded to a two-fold axis found along the *c* axis. The batch scale factor refined to 0.3680(16). The final refinement was performed using the HKL5 instruction (*i.e.*, the hkl file includes the set of reflections from domain 1 and the set of overlapped reflections from component 2). The two crystallographically independent molecules of the target compound were found to be ordered (Figure 2).

**Figure 3:**
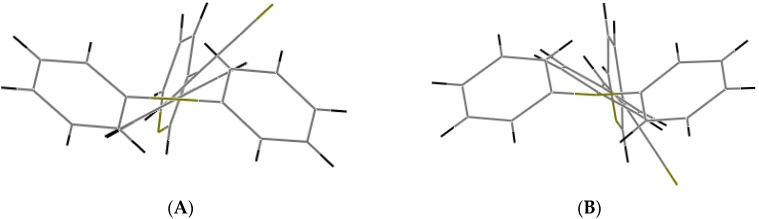
Two conformers **A** and **B** for the structure **I**.

**Figure 4:**
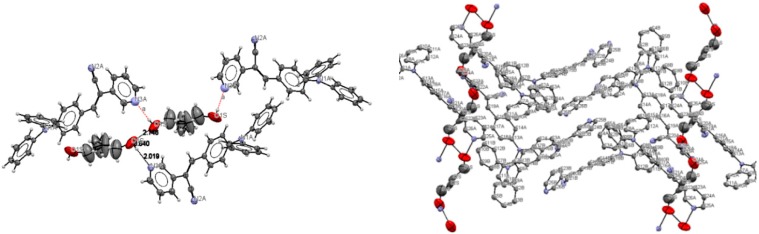
Intermolecular hydrogen bond interactions between the lattice solvent molecules (donor) and conformers **A** (acceptor).

The most interesting feature in the structure of **I** is concerned with the two independent conformers **A** and **B** (Figure 3). For **A**, there is one O-H(solvent)···N hydrogen bond interaction (Figure 4), and the -C≡N group [C(21A)-N(2A)] is found *anti* to the pyridine group. For **B**, there is no such O-H (solvent)···N interaction, and the -C≡N group [C(21B)-N(2B)] is found *syn* to the pyridine group. The molecules **A** and **B** are different conformers along the bond connecting the phenyl, double bond and pyridyl moeties. Table 2 provides a list of torsion angles for molecules **A** and **B**, which notable differ in sign. The *anti* conformation is typically found in different isomeric compounds that have already been reported in the literature [28,29,30], and also in the structures of **II** and **III** reported herein.

Lattice solvent ethanol molecules act as H-bond donor bridges between molecules of conformers **A** along the c axis. Table 2 provides a list of H-bond interactions in the structure of **I**.

The N3A···O1S and O1S···O1S intermolecular distances are *ca.* 2.77 and 2.75 Å, respectively, which suggests that the H atom from the -OH group of the ethanol molecules is disordered (this is not surprising as the ethanol molecules are most likely disordered) [45]. The presence of lattice solvent molecules in the crystal packing of **I** might indicate that crystal growth is optimized with O-H(solvent)···N(compound).

Table 3 includes a list of selected bond lengths and torsion angles for conformers **A** and **B**. The values for the pyridine ring and the *p*-(diphenylamino)phenyl group indicate that the *N*,*N*-diphenyl substituents are twisted through the single bonds of the N-atom, and those conformers are oppositely twisted. This behavior does not occur for structure **II** and for similar structures recently reported [28,29,30,31]. Table 3 also provides a list the selected bond lengths for **III**, as well as, the dihedral angles between the acrylonitrile linkage and the phenyl ring and with the diphenylaminophenyl or carbazole moieties.

**Figure 5:**
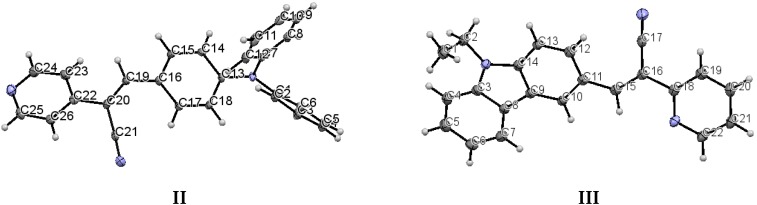
Displacement ellipsoid plots (50% probability level) **II** and **III**. Both compounds have *anti* conformation.

When the nitrogen atom is found at the *meta* position, the crystal packing significantly differs from those of the analogous structures without nitrogen and those of the structures of **II** (*para* position) and **III** (*ortho* position). The compounds **II** and **III** (Figure 5) have the *Z*-geometry about the ethylene bridge that links the aromatic rings and heterocyclic groups.

**Figure 6:**
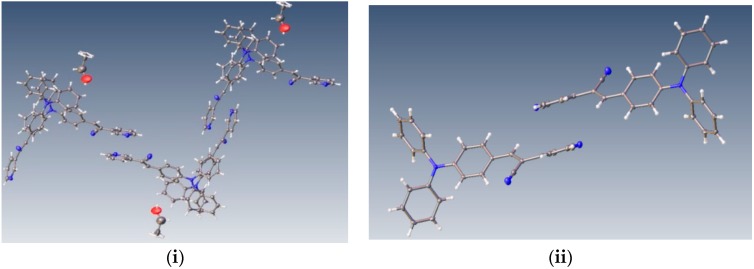
Perspective view of the packing structure of **I** (**i**) and **II** (**ii**) showing the strong π-π stacking interactions.

Understanding the different crystal packing modes (e.g., from herringbone to a co-facial π-stacking motif) is of importance to confer good electronic transfer properties. The molecules in **I** and **II** showed a tilted face-to-face arrangement (Figure 6) for the aromatic ring constituted of the pyridine ring with a centroid-centroid distance of 3.71 Å, a shift distance of 1.46 Å for **I**, and 3.84 Å of centroid-centroid distance and a shift distance of 1.843 Å for **II**. These values for the π-π interactions in the structures were automatically found by the program OLEX 2 [46].

These distances are typical for π**-**π aromatic face-to-face interactions with centroid distances > 3.65 Å and offsets in the range of 1.6–1.8 Å [47] and they reflect the distribution of electronic density that minimizes the π-electrons. However, for the structure of **III**, as well as several crystal structure reported with the -CH=CCN- moiety, no obvious π-π interactions are found, even though isomers were present. These packing motifs may be attributed to CH···π, edge-to-face interactions and they tune the packing structure from a herringbone mode toward more of a face-to-face π-stacking motif. Holmes *et al.* reported that in order to favor face-to-face π-stacking, the CH···π interactions should be minimized [18,22,48,49]. The packing of structures **I** and **II** showed that the pyridine unit, the position of the nitrogen atom within the pyridyl core, and the *p*-(diphenylamino)phenyl- group play important roles as well as the solvent used for crystallization.

**Figure 7:**
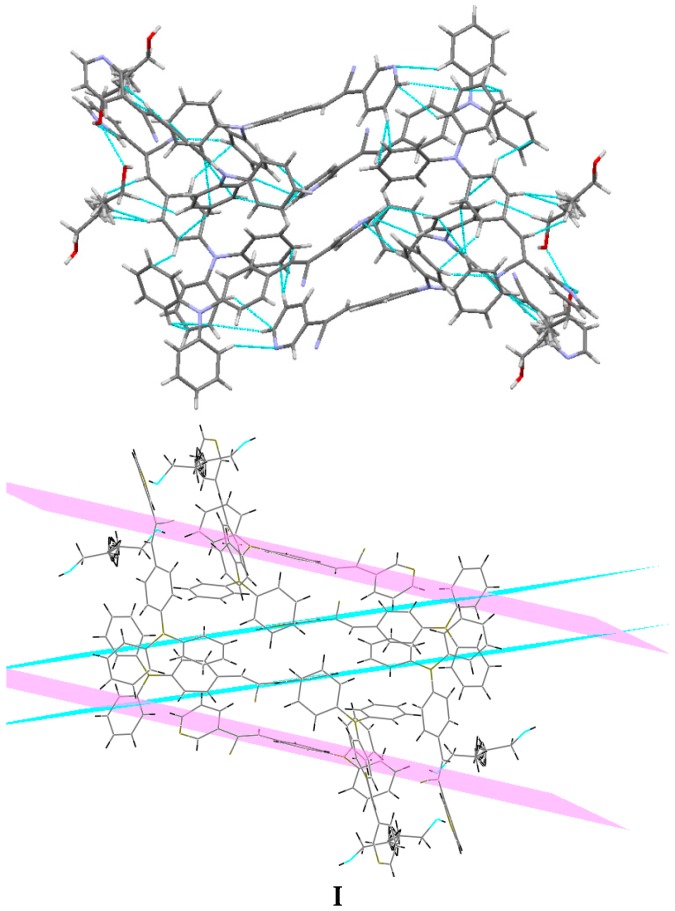

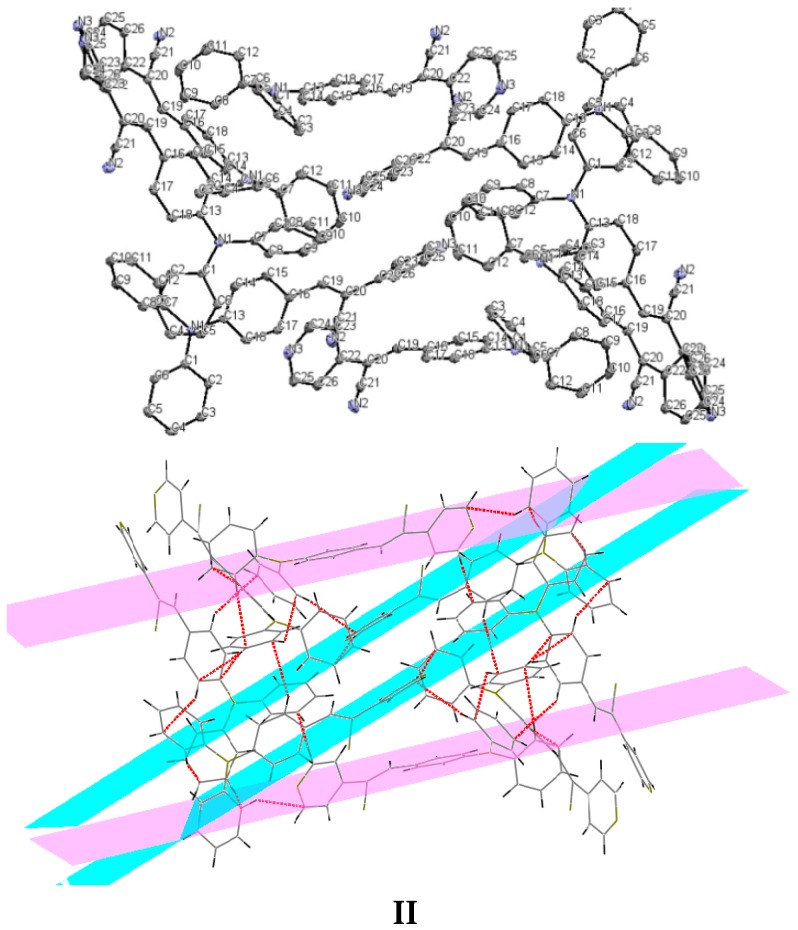
Crystal packing views of **I** and **II** with π-π stacking interactions occurring between neighboring aromatic rings.

**Figure 8:**
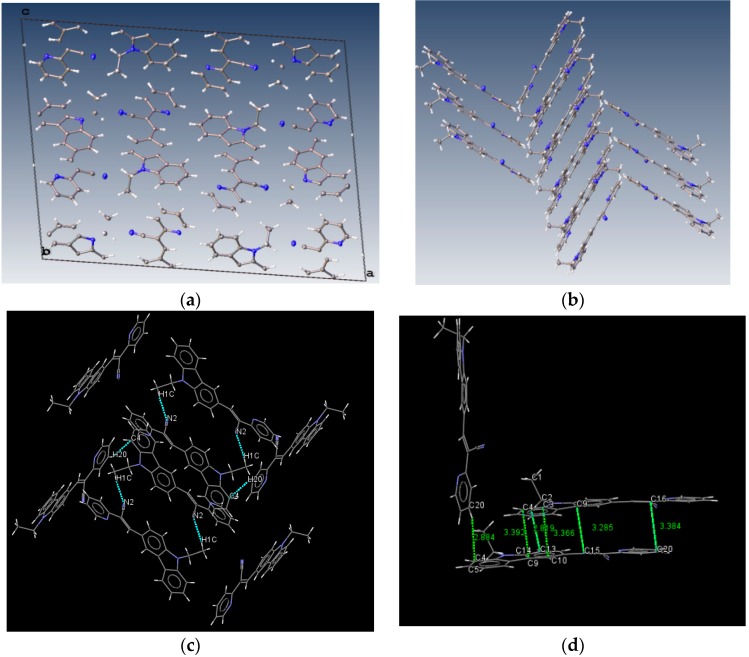
Molecule arrangement in the unit cell (**a**) and the herringbone packing motif (**b**) with the stacking of **III**, and a view showing the interactions, dashes lines (**c**) short contacts values along b axis (**d**).

Bao and coworkers reported that tetracene substituted with halogen atoms at the 5 and 11 positions adopted a face-to-face π-stacking motif with enhanced charge transport [22]. In these three packing modes, the charge transport could be three-dimensionally anisotropic and the highest mobility would be observed along the major π-stacking direction (Figure 7). In contrast, the lowest mobility would be found along the directions in which the molecules were insulated from one another. It is worth mentioning that the packing structure of **III** presents short contacts between the carbazole and the pyridine group. The interactions maintaining the structure of **III** are CH/EtCz···-C≡N of 2.74Å, and CH/(py)·····CH/π(Cz) of 2.88 Å (Figure 8); however, there are no strong π-π stacking interactions present from centroid-to-centroid, such as in **I** and **II**, as it can be seen the short contacts values for **III** (Figure 8d) calculated by Mercury software [50].

**Figure 9:**
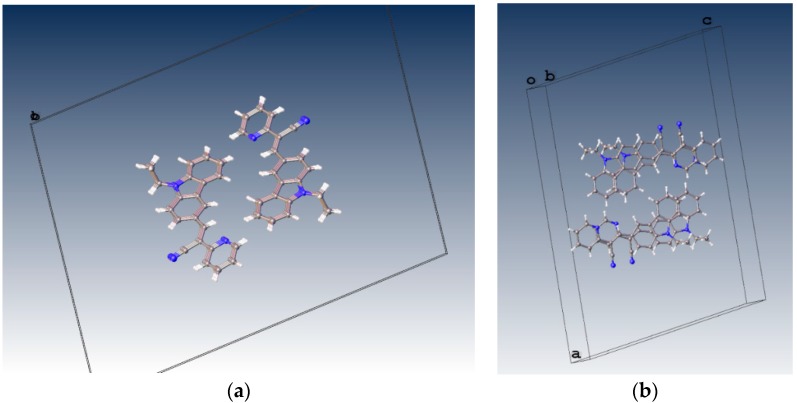
Molecular packing of **III** in the unit cell view from b axis (**a**) and from c axis (**b**).

From the Figure 8d it could be supposed that typical π-π interactions are present. However, in Figure 9 the distance between a pyridine plane and neighboring molecule indicated that there is not a cofacial π-π interaction arising a π-π overlap as it is exhibited in **I** and **II**. Consequently, the packing motif seems a classical herringbone packing with a strong slipped π-stacking indicating a weak π-π overlap between neighbor molecules [51].

**Figure 10:**
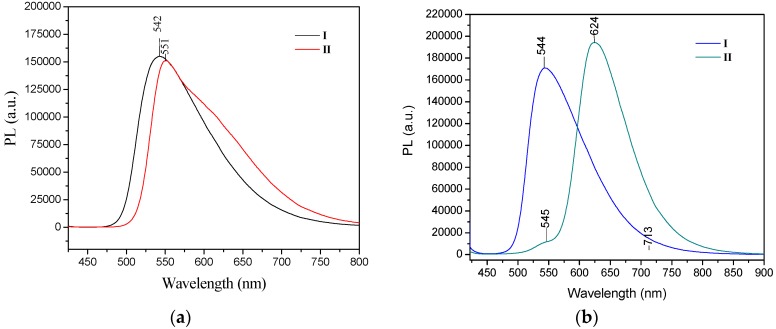
PL spectra of compounds **I**–**II** in (**a**) powder and (**b**) in crystal forms. Excitation wavelength for both conditions was 405 nm.

The UV/Vis absorption spectra recorded in chloroform for **I**, **II** showed similar maxima of absorption bands, one in the range 416–427 nm in solution with CHCl_3_ and a smaller absorption band at 297–301 nm. For the solid form, only the change for compound **II** is reported, which underwent a bathochromic effect of 24 nm (λ_max_ = 451nm) [52]. Only in compound **II** did the absorption spectrum display an obvious bathochromic shift in the solid state, which some authors suggest is due to the molecules adopting a π-aggregated form in the solid state [18]. The extended delocalization of the nitrogen electron pair onto the two phenyl rings from -N(Ph)_2_ accounts for the red shift of the absorption band. The emission spectra of **I** and **II** in chloroform solution in both the crystal and powder forms showed high fluorescence intensity, both in solution and in the solid state. The photoluminescence spectra of **I** and **II** were compared to reported values [52]. Crystals of **II** showed a strong bathochromic effect of almost 80 nm as crystals, with λ_em_ at 624 nm. However, the fluorescence emission maxima for compound **I** were almost the same for the powder form (535 nm) and the crystals (544 nm) (Figure 10).

**Figure 11:**
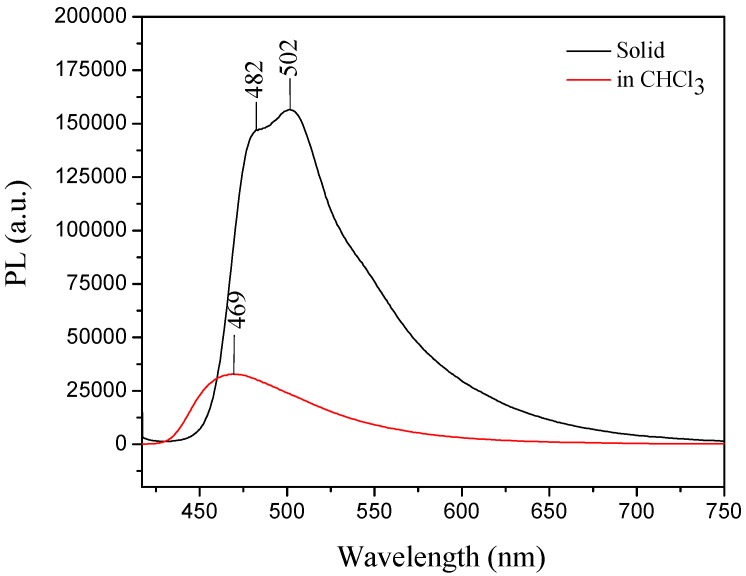
Emission of compound **III** in solid form (−) and in solution (−) indicating that **III** exhibited the AIE property. Excitation wavelength for both conditions was 405 nm.

For **III**, the UV-vis absorption wavelength maximum in solution was at 380 nm, whereas in the solid state its absorption maximum (λ_max_) was 398 nm (**III**) [30]. The compound showed a typical AIE effect, because the emission maximum in the solid form was observed at 502 nm and the emission in solution was weak, which could be attributed to the carbazole group effect [30,53] (Figure 11).

### 2.2. Electrochemical Properties.

**Figure 12:**
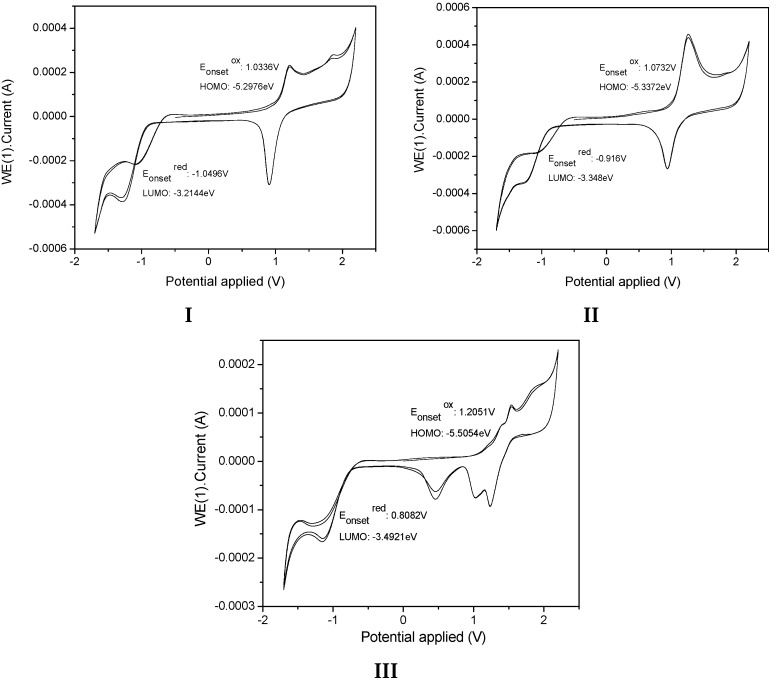
Cyclic voltammograms of **I**, **II** and **III** in 1 mM in 0.1M TBAFP_6_/CH_2_Cl_2_ solution with Ag/AgCl as reference electrode and a Pt wire as the working electrode. A scan rate of 50 mVs^−1^ was used.

The electrochemical characteristics of the samples **I**, **II**, and **III** were investigated by cyclic voltammetry (CV) (Figure 12). The onset oxidation ($E_{\mathrm{Ox}}^{\mathrm{onset}}$) estimated from CV curves for **I**, **II**, and **III** are summarized in Table 4. We determined the HOMO and LUMO energy levels of the samples, as calculated from the CV tests applying published equations [54] and using ferrocene as the external standard. All measurements were carried out at room temperature.

$$HOMO=-(E_{ox}-E_{1/2\left(ferrocene\right)}+\;4.8eV)$$

$$LUMO=-\left(E_{red}-E_{1/2\left(ferrocene\right)}+4.8eV\right)$$

The value of **III** has lower energy HOMO and LUMO levels, suggesting a lower electron injection barrier than observed for **I** and **II**, indicating that **III** had a stronger tendency to donate an electron, whereas the high LUMO energy level of **II** indicated its strong electron-withdrawing ability. The electronic energy gap values were estimated to be 2.08 eV, 1.99 eV, and 2.01eV for **I**, **II** and **III**, respectively (Table 4). We also determined the energy gap values, as calculated from the absorption spectrum in CHCl_3_ by using ΔE^opt^ = *hc*/λ (ΔE^opt^ (eV) =1237.5/λ(nm).

The energy of the lowest gap, which results from a more extended conjugation, was attributed to the (4-diphenylamino) molecule moiety in **II**. However, the values indicated that the position of substitution of the pyridyl unit reduced the molecule slightly, making it more easily oxidizable. In the structure, the electron withdrawing effect from the pyridine group could also affect the conjugation structure.

### 2.3. DFT Calculations

We carried out the Gaussian09 program optimization calculations with the M06L method using the cc-pVDZ basis set in the gas and solvent phases.

Geometries with small root-mean-square deviations were obtained with respect to the crystallographic data (the largest 8 degrees in dihedral angles). The frontier HOMO and LUMO energies and gap energies were evaluated in the different solvents (Table 5). The effect of solvent was evaluated by the PCM method. The most energetically stable structures were obtained in solutions of methanol and dimethyl sulfoxide. Also, the highest values of dipole moment (µ) and the lowest were correlate to the solvent polarity used to obtain suitable crystals for X-ray characterization (see Table 6) [55,56,57,58].

**Figure 13:**
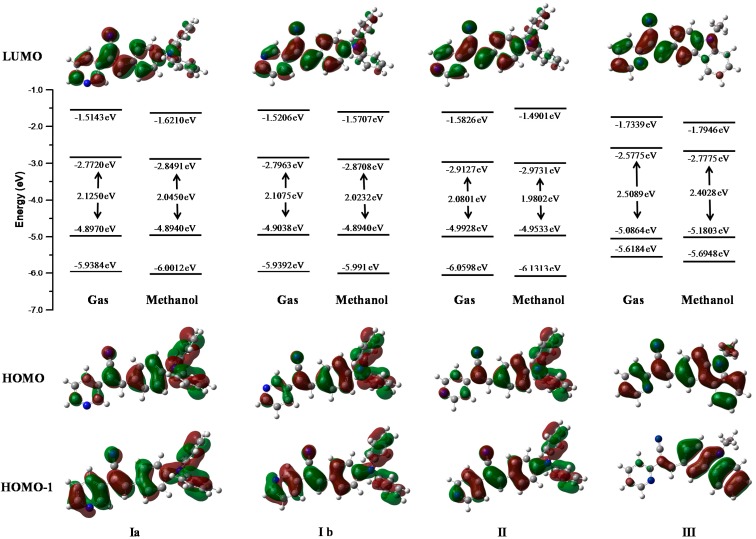
Molecular orbitals of compounds obtained in gas and methanol and phases at M06L/cc-pVDZ theory level.

Figure 13 represents the energies of the molecular orbitals LUMO+1, LUMO, HOMO, HOMO-1 in the gas phase and in one of the most stable solvent phase (methanol). Isosurfaces in the diagram represent the maps of HOMO and HOMO-1(bottom) and LUMO (top). The character and energy levels of HOMOs and LUMOs (and the corresponding energy gaps) were determined on fully optimized geometries and compared with electrochemical measurements. The theoretical HOMO energy levels of the three compounds **I**, **II** and **III** in the gas phase were close together, −4.89, −4.99 and −5.08 eV, respectively. **I** and **II** *p*-diphenylamino substituents caused a greater delocalization on the phenyl core than the pyridyl ring. The theoretical calculation showed a delocalization on the pyridine-carbazole backbone for **III**. Thus, the character of the HOMO of **I** and **II** were very different from that of **III**, for which the HOMO presented a clear pyridyl-phenyl-carbazole delocalization leading to the highest HOMO level (−5.08 eV).

The present calculations were almost in accordance with electrochemical data (Table 4), which led us to conclude a better delocalization in **I**, **II** and **III,** therefore diminishing the HOMO level from **I**, **II** to **III** −5.29, −5.33 to −5.50 eV. With respect to the LUMO levels of the pyridine-substituted **I**, **II** and **III**, they were calculated to be between −2.77, 2.91 and −2.57 eV, respectively, presenting a pyridyl character without contribution of the *p*-diphenylamino and carbazole moieties. The LUMO energy levels of **II** are 0.34 eV higher than that of **III** (−2.57eV) showing the less intense withdrawing effect of the pyridyl unit compared to the carbazole unit. The LUMO level of **III** was calculated at −2.57 eV, lower than that of **I** and **II** and the decrease of the LUMO in **II** emphasized the withdrawing effect of the pyridyl unit and its efficient conjugation with the carbazole unit, rendering this molecule the most easily reducible. The main tendency of the LUMO levels obtained through theoretical calculations did not agree with our electrochemical conclusions, with the lowest LUMO recorded for **I**. The LUMO calculated for **I**, **II** and **III** are close together (0.44eV), and their LUMO values determined through electrochemical measurements were also very similar (−3.21, −3.34, and −3.39 eV, respectively).

The theoretical energy gaps of **I**, **II** and **III** were close to 2.12, 2.08, and 2.50 eV, respectively, values closer to the energy gap values obtained from the electrochemical measurements (2.08 eV for **I**, 1.98 for **II**, and 2.01 for **III**) showing a good correlation between theoretical calculations. Furthermore, the values showed better fit with the theoretical calculations when the polarity of the solvent was increased.

## 3. Experimental Section

The compounds **I**–**III** were synthesized according to the reported methods [30,59]. We utilized several synthetic procedures to obtain adequate crystals of **I**–**III** to permit single crystal characterization (Table 6). In some cases, the crystals were too small to allow diffraction and in other procedures, the compounds were almost insoluble in the solvents. The majority of the isomers were crystallized after reaction or were purified by crystallization.

The optimum crystallization procedures for each compound are indicated below. For **I**, the solid (0.028 g) was dissolved in ethanol:cyclohexane (12 mL) 58 °C and the vessel was set aside at 4 °C and after 3 days, yellow crystals were obtained. Compound **II** (0.035 g) was dissolved in dimethylsulfoxide (DMSO) at 90 °C; the vessel was set aside at room temperature and after 9 days, yellow crystals were formed. Compound **III** (0.020 g) was dissolved in cyclohexane (15 mL) at room temperature, the vessel was set aside at 4 °C and after 4 days, tiny yellow crystals were obtained.

### 3.1. Single Crystal X-ray Diffraction (SCXRD)

All reflection intensities for **I** and **II** were measured at 110(2) K (after the crystals were flash cooled from RT) using a KM4/Xcalibur (detector: Sapphire3) with enhance graphite-monochromated Mo Kα radiation (λ = 0.71073 Å) apparatus under the program CrysAlisPro (Versions 1.171.35.11 or 1.171.36.24, Agilent Technologies 2012, Santa Clara, CA, USA). Data for **III** were collected at 110(2) K using a SuperNova diffractometer (equipped with Atlas detector) with Cu *K*α radiation (λ = 1.54178 Å) under the program CrysAlisPro (Version 1.171.36.28, Agilent Technologies 2013). The same program was used to refine the cell dimensions and for data reduction. The structures were solved with the programs SHELXS-97/SHELXS-2013, and were refined on *F*^2^ with SHELXL-97/SHELXL-2013 [60]. Analytical numeric absorption corrections based on a multifaceted crystal model were applied using CrysAlisPro. The temperature of the data collection was controlled using the system Cryojet (Oxford Instruments, Abingdon, Oxford, UK). The H atoms were placed at calculated positions using the instructions AFIX 23, AFIX 43, AFIX 123, AFIX 137 or AFIX 147 with isotropic displacement parameters having values 1.2 or 1.5 times *U*eq of the attached C or O atoms.

### 3.2. Absorbance and Emission (UV-VIS and PL)

The absorbance spectra were acquired on a SD2000 spectrometer (Ocean Optics, Dunedin, FL, USA) equipped with a pulse Xenon light source P-2 (Ocean Optics for the UV region (220–270 nm) and a Cary 300 Spectrometer (Agilent) equipped with a deuterium and halogen lamp. The wavelength detection range was 190–900 nm. For measurements in solution, the solvents were of spectroscopic grade and were preliminarily checked for the absence of absorbing or fluorescent impurities within the scanned spectral ranges. For powder samples, the absorption was measured using pellets prepared with KBr. A UV/Vis DT 1000 CE light source (Analytical Instrument Systems, Inc., Flemington, NJ, USA) was used for measuring absorption. Emission spectra (PL) were acquired from a QE-Pro-FL (Ocean Optics) equipped with a laser diode excitation source at a wavelength of 405 nm.

### 3.3. Cyclic Voltammetry (CV)

Measurements were carried out with a PGSTAT128N-serial no. AUT85577 potentiostat (Keysight, Santa Rosa, CA, USA) using a three electrode cell assembly comprised of Ag/Ag⁺ in solution of 3 M KCl as the reference electrode, a platinum wire (ϕ = 0.2 mm) as working electrode and counter-electrode, using a solution in CH_2_Cl_2_ containing 0.1 M supporting electrolyte of tetrabutylammonium hexafluorophosphate (TBAFP_6_), and ferrocene was used as external standard. The scanning rate was 50 mV/s.

## 4. Conclusions

In this work, we have reported three molecular structures of α,β-unsaturated acrylonitriles which crystallized into single crystals with different *Z* values and according to crystallography data gave information about the arrangement packing that is possible to correlate with the emission with a bathochromic shift. Such emission enhancement in the aggregate state is due to strong π-π molecular stacking in the crystal lattice, which is does not depend on of intermolecular H-bonding interactions between molecule-solvent because it is present in both **I** and **II**. The results from crystal structures with Z′ > 1 represent a possible solution to the problem of packing molecules in three dimensions, because the three molecules **I**, **II** and **III** have eight molecules per unit cell, which could be due to the crystallization process, as is particularly well illustrated with **I** as an example where the compound adopts high Z′ or high symmetry. Also the systems reported for **I** and **II** showed stacking via π-π interactions with values closer to those typically found for geometrical parameters of aromatic π**-**π interactions centroid distances. The data for **I** represented a rare case with complete hydrogen-bonding of the chains as **I**, whose structure was all the more remarkable because of the presence of both the *syn* and *anti* conformers at the double bond. One of the ultimate goals of solid-state chemistry is the ability to predict or computationally calculate the experimental crystal structure(s) of a compound solely from knowledge of its molecular structure. This factor is a remarkable problem in the computational field because the possible occurrence of more than one symmetry unique molecule greatly complicates the crystal.

## Figures and Tables

**Table 1:** Summary of Crystallography Data for **I**–**III**.

Empirical Formula	I	II	III
	C_26_H_19_N_3_, 0.395 (C_2_H_6_O)	C_26_H_19_N_3_	C_22_H_17_N_3_
Color, habit	yellow, irregular shape	yellow, plate	yellow, thin needle
Crystal system	Monoclinic	Orthorhombic	Monoclinic
Formula weight	391.68	373.44	323.38
Space group	*P2/c*	*Pbcn*	*C2/c*
T (K)	110(2)	110(2)	110(2)
A (Å)	24.9448(10)	24.4603(9)	30.4066(6)
b (Å)	10.5094(5)	10.1791(3)	4.81650(10)
c (Å)	15.4015(6)	15.4796(6)	22.6451(5)
α (°)	90	90	90
β (°)	94.300(4)	90	98.965(2)
γ (°)	90	90	90
V (Å^3^)	4026.2(3)	3854.2(2)	3275.94(12)
*Z*, *Z′*	8, 2	8, 1	8, 1
D_c_ (g·cm^−3^)	1.292	1.287	1.311
F (000)	1650.4	1568	1360
μ (mm^−1^)	0.078	0.077	0.612
λ (Å)	0.71073	0.71073	1.54178
Crystal size (mm^3^)	0.44 × 0.20 × 0.08	0.25 × 0.21 × 0.06	0.73 × 0.06 × 0.063
2θ_max_ (°)	48.6	50	134.5
N_total_, N_unique_	23,831, 7364	17,555, 3395	9198, 2944
N_obs_ (I > 2.0 σ(I))	5257	2671	2497
R1 (I > 2.0 σ(I))	6.10	4.23	3.66
wR^2^ (I > 2.0 σ(I))	13.77	8.94	8.72
goodness-of-fit	1.055	1.028	1.045
Largest diff peak and hole (e·Å^−3^)	0.61 and −0.57	0.19 and −0.21	0.22 and −0.21

**Table 2:** Hydrogen-bonds D···H···A (Å) and D−H⋯A angles (°) of compound **I**.

D	A	Symmetry for A	d(D···A)	D(A···H)	D(D-H)	D-H···A
O1S	O1S	x, 1 − y,−1/2 + z	2.746	-	-	101.21
O1S	N3A	x, y, −1 + z	2.770	N(3)······H(1S) 2.019	O(1S)-H(1S) 0.8400	148.57

**Table 3:** Bond lengths (Å) and torsion angles (°) selected in the crystal structures of **I** and **II**.

Bond Length	I		II	III
	A	B		
N(1)-C(1)	1.429(5)	1.429(5)	1.424(2)	
N(1)-C(2)				1.522(2)
N(1)-C(3)				1.3910(17)
N(1)-C(7)	1.431(5)	1.409(5)	1.4279(19)	
N(1)-C(13)	1.397(5)	1.407(5)	1.4042(19)	
N(1)-C(14)				1.3800(18)
C(19)-C(20)	1.352(6)	1.339(6)	1.348(2)	
C(19)-C(16)	1.459(6)	1.456(6)	1.454(2)	
C(15)-C(11)				1.449(2)
C(15)-C(16)	1.365(6)	1.386(6)	1.398(2)	1.351(2)
C(16)-C(17)	1.397(6)	1.390(6)	1.404(2)	1.4381(19)
C(16)-C(18)				1.483(2)
C(20)-C(22)	1.483(6)	1.476(6)	1.487(2)	
C(23)-C(22)	1.393(6)	1.394(6)	1.392(2)	
C(17)-N(2)				1.1493(19)
C(18)-N(3)				1.3460(18)
C(22)-N(3)				1.3318(19)
C(26)-N(3)	1.330(6)	1.333(6)		
C(20)-C(21)	1.429(6)	1.447(5)	1.437(2)	
C(21)-N(2)	1.147(6)	1.135(5)	1.152(2)	
C(25)-N(3)	1.331(6)	1.333(6)	1.336(2)	
C(24)-N(3B)			1.341(2)	
Torsion angle				
C(14)-C(13)-N(1)-C(7)	150.0(4)	−154.6(4)	30.0(2)	
C(14)-C(13)-N(1)-C(1)	−31.3(6)	35.6(6)	−148.58(15)	
C(8)-C(7)-N(1)-C(1)	−41.5(5)	39.7(5)	46.5(2)	
C(12)-C(7)-N(1)-C(13)	−42.7(6)	48.4(6)	48.6(2)	
C(18)-C(13)-N(1)-C(1)	138.5(4)	−144.8(4)	31.9(2)	
C(18)-C(13)-N(1)-C(7)	−30.1(6)	24.9(6)	−149.59(15)	
C(2)-C(1)-N(1)-C(13)	−45.0(5)	36.8(6)	37.5(2)	
C(21)-C(20)-C(22)-C(23)	−30.0(6)	−152.4(4)	−151.57(15)	
C(16)-C(19)-C(20)-C(22)	−179.0(4)	−178.6(4)	−178.98(15)	
C(15)-C(16)-C(19)-C(20)	−12.7(8)	22.2(7)	−161.32(17)	
C(16)-C(19)-C(20)-C(21)	−2.8(8)	4.4(7)	6.9(3)	
C(19)-C(20)-C(22)-C(23)		30.3(6)	33.9(2)	
C(19)-C(20)-C(22)-C(26)	−34.7(7)	−146.6(4)		
C(21)-C(20)-C(22)-C(26)		30.7(6)	28.9(2)	
C(17)-C(16)-C(19)-C(20)			20.5(3)	
C(17)-C(16)-C(18)-C(19)				1.68(19)
C(11)-C(15)-C(16)-C(17)				−3.1(2)
C(12)-C(11)-C(15)-C(16)				−13.8(2)
C(15)-C(16)-C(18)-N(3)				−1.53(19)
C(10)-C(11)-C(15)-C(16)				167.60(14)
C(13)-C(14)-N(1)-C(3)				178.78(14)
C(4)-C(3)-N(1)-C(2)				1.7(2)

**Table 4:** Electronic properties **I**, **II** and **III**.

	Cyclic voltammetry					Theoretical calculations			Optical data
	$E_{\mathrm{Ox}}^{\mathrm{onset}}$ (V)	$E_{\mathrm{Red}}^{\mathrm{onset}}$ (V)	HOMO (eV)	LUMO (eV)	ΔE (eV)	HOMO (eV)	LUMO (eV)	ΔE (eV)	ΔE^opt^ (eV)
**I**	1.0336	−1.0496	−5.2976	−3.2144	2.0832	−4.897000	−2.772046	2.124954	2.68
**II**	1.072	−0.916	−5.3372	−3.348	1.9892	−4.992784	−2.912730	2.080055	2.72
**III**	1.2051	−0.8082	−5.5054	−3.4921	2.0133	−5.086392	−2.577483	2.508910	2.63

**Table 5:** Theoretical data of **I**, **II** and **III** in gas and solvent phases at M06L/cc-pVDZ theory level.

HOMO energy (eV)								
Compound	Gas	C_6_H_12_	CHCl_3_	THF	CH_2_Cl_2_	C_2_H_5_OH	CH_3_OH	DMSO
**A**	−4.8970	−4.8837	−4.8856	−4.8880	−4.8891	−4.8932	−4.8940	−4.8945
**B**	−4.9038	−4.8886	−4.8883	−4.8899	−4.8907	−4.8935	−4.8940	−4.8945
**II**	−4.9928	−4.9664	−4.9560	−4.9544	−4.9541	−4.9533	−4.9533	−4.9533
**III**	−5.0864	−5.1117	−5.1446	−5.1579	−5.1623	−5.1781	−5.1803	−5.1827
LUMO energy (eV)								
Compound	Gas	C_6_H_12_	CHCl_3_	THF	CH_2_Cl_2_	C_2_H_5_OH	CH_3_OH	DMSO
**A**	−2.7720	−2.7965	−2.8237	−2.8338	−2.8368	−2.8474	−2.8490	−2.8507
**B**	−2.7963	−2.8197	−2.8461	−2.8556	−2.8588	−2.8692	−2.8708	−2.8722
**II**	−2.9127	−2.9326	−2.9541	−2.9617	−2.9639	−2.9720	−2.9731	−2.9742
**III**	−2.5775	−2.6455	−2.7130	−2.7380	−2.7459	−2.7734	−2.7775	−2.7813
Gap energy (eV)								
Compound	Gas	C_6_H_12_	CHCl_3_	THF	CH_2_Cl_2_	C_2_H_5_OH	CH_3_OH	DMSO
**A**	2.1249	2.0871	2.0618	2.0542	2.0523	2.0458	2.0449	2.0439
**B**	2.1075	2.0689	2.0422	2.0343	2.0319	2.0243	2.0232	2.0224
**II**	2.0800	2.0338	2.0019	1.9927	1.9902	1.9813	1.9802	1.9791
**III**	2.5089	2.4662	2.4316	2.4199	2.4164	2.4047	2.4028	2.4014
μ (Debye)								
Compound	Gas	C_6_H_12_	CHCl_3_	THF	CH_2_Cl_2_	C_2_H_5_OH	CH_3_OH	DMSO
**A**	4.6302	5.3812	5.9549	6.1395	6.1962	6.3849	6.4112	6.4370
**B**	7.0370	8.0966	8.9946	9.3050	9.4029	9.7378	9.7858	9.8333
**II**	7.1955	8.3345	9.2504	9.5546	9.6490	9.9654	10.0098	10.0535
**III**	3.3975	3.9375	4.3492	4.4809	4.5212	4.6546	4.6731	4.6913

**Table 6:** Solvent conditions tested to obtain tiny crystals.

I	II	III
Ethylacetate:hexane (1:2)	Ethylacetate:hexane (1:2)	Ethylacetate:hexane (1:2)
Ethanol–cyclohexane (1:3)	Acetone:water (1:1)	Ethanol-cyclohexane 1:3
Ethanol–DMSO 1:5	Ethanol:DMF 1:5	Ethanol-DMSO 1:5
Ethanol:cyclohexane (3:2)√	DMSO√	Cyclohexane√

√ = tiny crystals too small to characterize.

## Supplementary Materials

Crystallographic data (excluding structure factors) reported in this paper have been deposited with the Cambridge Crystallographic Data Centre as supplementary publication no. CCDC 1006818, 1006819 and 1006820. Copies of available material can be obtained, free of charge, on application to the CCDC, 12 Union Road. Cambridge CB2 IEZ, UK, (fax: +44-(0)1223-336033 or e-mail: deposit@ccdc.cam.ac.uk).
